# Advances and Applications of Lung Organoids in the Research on Acute Respiratory Distress Syndrome (ARDS)

**DOI:** 10.3390/jcm13020346

**Published:** 2024-01-08

**Authors:** Xingwu Zhang, Longxiang Su, Pan Pan

**Affiliations:** 1 College of Pulmonary & Critical Care Medicine, 8th Medical Center, Chinese PLA General Hospital, Beijing 100091, China; zhxiwu@163.com; 2 School of Medicine, Tsinghua University, Beijing 100084, China; 3 Department of Critical Care Medicine, State Key Laboratory of Complex Severe and Rare Diseases, Peking Union Medical College Hospital, Peking Union Medical College, Chinese Academy of Medical Science, Beijing 100730, China

**Keywords:** Acute Respiratory Distress Syndrome (ARDS), lung organoid, pluripotent stem cell, microfluidic system

## Abstract

Acute Respiratory Distress Syndrome (ARDS) is a sudden onset of lung injury characterized by bilateral pulmonary edema, diffuse inflammation, hypoxemia, and a low P/F ratio. Epithelial injury and endothelial injury are notable in the development of ARDS, which is more severe under mechanical stress. This review explains the role of alveolar epithelial cells and endothelial cells under physiological and pathological conditions during the progression of ARDS. Mechanical injury not only causes ARDS but is also a side effect of ventilator-supporting treatment, which is difficult to model both in vitro and in vivo. The development of lung organoids has seen rapid progress in recent years, with numerous promising achievements made. Multiple types of cells and construction strategies are emerging in the lung organoid culture system. Additionally, the lung-on-a-chip system presents a new idea for simulating lung diseases. This review summarizes the basic features and critical problems in the research on ARDS, as well as the progress in lung organoids, particularly in the rapidly developing microfluidic system-based organoids. Overall, this review provides valuable insights into the three major factors that promote the progression of ARDS and how advances in lung organoid technology can be used to further understand ARDS.

## 1. Basis and Progress in Acute Respiratory Distress Syndrome

### 1.1. Introduction

According to the widely-accepted 2012 Berlin definition [1], Acute Respiratory Distress Syndrome (ARDS) is an acute onset of lung injury characterized by bilateral pulmonary edema, diffuse inflammation, hypoxemia with a low P/F ratio, and one exclusion criterion of cardiac causes. Systematic inflammation (non-pneumonia sepsis, pancreatitis, high-risk surgery, and non-cardiogenic shock, etc.) and pulmonary local injury (bacterial, viral, and other atypical types of pneumonia, etc.) are the two major causes of ARDS. High incidence, morbidity, and high medical expenditure make ARDS one of the most prevalent and important complications in the intensive care unit (ICU) [2]. Mortality varies from 34.9% to 46.1% in mild to severe ARDS patients. In addition, as a serious concern in the new era, the COVID-19 pandemic has led to updates in the definition of ARDS, which shows a longer onset of process [3], normal or even high lung compliance [4], and heavier dependence on mechanical ventilation [5].

The central and classic pathological feature of ARDS is diffuse alveolar damage (DAD), which not only is caused by excessive inflammation but is also the reason for diffuse inflammation [6]. Where DAD is concerned, epithelium and endothelium injuries are considered the central processes in pulmonary ARDS and extrapulmonary ARDS, respectively, although it is difficult to differentiate between these two conditions in clinical practice [7,8]. Apart from DAD and systematic inflammation, mechanical stress is a vital factor contributing to the progression of ARDS. While ventilators are acknowledged as life-saving, they can also exacerbate ARDS. Accordingly, several mechanical ventilation approaches have been identified to partly alleviate these adverse effects [9]. However, despite efforts to elucidate its pathophysiological mechanisms, the current understanding of ARDS is incomplete due to the limitations of in vivo and in vitro models. Here in this review, we will focus on the three major factors (epithelium injury, endothelium injury, and mechanical stress) that promote the progression of ARDS and discuss the important discoveries and remaining issues in the existing research.

### 1.2. Epithelium Injury in ARDS

It has been proven that alveoli are composed of two types of epithelial cells: type I and type II alveolar epithelial cells (AT1 and AT2 cells) [10,11]. It has long been established that AT2 cells serve as the progenitor of AT1 cells, although recent findings suggest that at least one subtype of AT1 cells (Hopx^+^Igfbp2^−^) may also have the capacity to trans-differentiate into AT2 cells [12]. Due to their high proliferative capacity, AT2 cells are easier to culture in vitro. In contrast, AT1 cells are not only difficult to maintain in primary culture but also to observe under a light microscope, thereby limiting both explorative and conclusive research on AT1 cells in any kind of lung injury [13,14].

AT1 cells’ primary roles are to facilitate gas exchange, maintain the structural integrity of sacs, and stabilize ion and fluid balance. Furthermore, AT1 cells have also been found to have immunological functions. For example, Yamamoto et al. found AT1 cells as a novel source of CXCL5 after LPS stimulation and upregulation of TLR2 and STING, the latter of which mediates innate immunity for the recognition of bacterial DNA and expression of type I IFNs [15]. Additionally, Lin et al. suggested that the membrane raft structure displayed on AT1 cells is necessary for the passage of paracellular neutrophils into the alveoli [16]. Conversely, AT1 cells can also be the victims of diffuse alveolar injury, with dysfunction correlated with diffuse edema and resultant hypoxemia [17,18].

The basic functions of AT2 cells include producing and secreting surfactant proteins, expression of immunomodulatory proteins, balancing the transepithelial water movement, and replenishment of AT1 cells after injury [14]. In the progression of ARDS, AT2 cells are the central mediators of injury to DAD. Flooding of alveoli due to decreased surfactant phospholipids, as a consequence of IAV infection-induced abnormality of lamellar bodies in AT2 cells, is the primary cause of atelectasis and reduced pulmonary compliance [19,20,21]. Additionally, Interferon-mediated crosstalk between AT2 and AT1 cells initiated by IAV infection leads to degradation and reduction of Na,K-ATPase, which further contributes to edema [22]. Hypercapnia alone in AT2 cells has been identified as an alternative trigger for Na,K-ATPase degradation [23]. ENaC, CFTR, and Na,K-ATPase dysfunction were found to be exclusive to IAV-infected cells rather than uninfected cells, wherein CFTR dysfunction persisted even after the period of infection [24]. In vitro studies using NCI-H441 and A549 cell lines revealed that IAV infection disrupts the epithelial tight junction, thus resulting in edema [25,26]. Nevertheless, considering the proportion of air-contacting area attributed by AT1 cells to alveoli, the disruption of the tight junction might be more relevant in AT1 cells.

Aside from their classical function, fully functional AT2 cells may also hinder the translocation of localized cytokines into circulation. In cases of uncontrolled ARDS, primary damage to injured epithelial cells is amplified via positive-feedback loops mediated by danger-associated molecular patterns (DAMPs) [27]. Interestingly, AT2 cells are also able to produce anticoagulants such as soluble thrombomodulin and endothelial protein C receptor (EPCR) and activate protein C and the anticoagulant pathway. However, pro-inflammatory cytokines can lead to inactivation of TM [28,29], EPCR [30], and tissue factor pathway inhibitor (TFPI) [31] through different mechanisms, which can result in fibrin deposition along the damaged alveolar surface.

After decades of research, many questions related to epithelial cell injury in ARDS have not been fully elucidated, such as the function of AT1 cells and their role in ARDS. Furthermore, there is limited research on the crosstalk between different types of epithelial cells and endothelial cells. It has also been acknowledged that epithelial cell injury can lead to several consequences, including surfactant depletion, diffuse edema, and a pro-coagulant state of local circulation, but little attention has been paid to the causal relationship among these consequences. Understanding the inter-relationship and sequence of different pathophysiological processes in ARDS is of great clinical significance, but research exclusively focusing on one or two cell types is insufficient to provide valuable insight.

### 1.3. Endothelium Injury in ARDS

Around 32% of all ARDS cases are derived from sepsis or other extrapulmonary injury, where alveolar damage is frequently driven by endothelial dysfunction and local inflammation [32]. Multiple factors contribute to endothelium damage, including circulating pathogens, the epithelium, and numerous pro-inflammatory cells. During the progression of sepsis, molecules and proteins released from damaged cells during infection and trauma, namely, damage-associated molecular patterns (DAMPs), play a central role in mediating circulating inflammatory factors and diffuse alveolar damage [33]. Sentinel immune cells are activated in DAMPs and produce multiple cytokines, which upregulate adhesion molecules of the endothelium to recruit and activate circulating and migrating neutrophils [34]. In addition, multiple signals lead to neutrophil adherence and inflammation, including circulating pro-inflammatory molecules (platelet-activating factor, angiopoietin 2, tumor necrosis factor, vascular endothelial growth factor, inflammasome product IL-1, and others) inflammatory mediators (interleukin (IL)-1, IL-6, IL-8, tumor necrosis factor-alpha (TNF-α)), death or pyroptosis of alveolar macrophages [35] and degradation of the endothelial glycocalyx [36]. Inflammation mediated by neutrophils is protective against infection but also injurious to bystander tissues through neutrophil extracellular traps (NETs) or other mechanisms [37].

There are also some atypical pathways mediating endothelium damage. For example, cell-free hemoglobin leads to increased paracellular permeability of human pulmonary microvascular endothelial cell monolayers through oxidative effects, which could be partially abrogated by acetaminophen [38]. On the other hand, Hough and his colleagues applied concentrated hydrochloric acid on the alveolar epithelium and generated life-threatening pulmonary edema, which is partially similar to that in typical ARDS. Interestingly, secondary endothelium injuries are not caused by concomitant membranes but by H_2_O_2_-mediated epithelial–endothelial paracrine signaling, and the subsequent endothelial cell damage is caused by uncoupling protein 2 (UCP2)-induced mitochondrial depolarization, suggesting UCP2 as a therapeutic target to block secondary damage caused by epithelial–endothelial paracrine signaling [39].

At present, there is already a relatively clear immunological process model of endothelial cell injury in the inflammatory process, including processes mediated by various immune cells and inflammatory mediators. However, endothelium damage in the pulmonary environment still needs further clarification, especially of the following aspects: (1) the cause of endothelial injury due to the interaction between endothelial cells and epithelial cells; (2) the microenvironment effect, including the causes of endothelial injury by a variety of cells and inflammatory mediators in ARDS; (3) the role of endothelial cells in the repair and recovery process of ARDS.

### 1.4. Mechanical Injury in ARDS

Ventilators are vital equipment in the treatment of ARDS and could also become a direct cause of ARDS [40]. Volutrauma, barotrauma, atelectotrauma, and biotrauma are the major aspects of injury caused by the ventilator, namely, ventilator-induced lung injury (VILI). Theoretically, high tidal volumes are the direct cause of volutrauma, while barotrauma describes pressure-related lung injury. It was noticed that pulmonary edema caused by volutrauma is not induced in animals with similar pressure but lower tidal volume, indicating an independent source of pulmonary injury [41]. On the other hand, damage caused by cyclical opening and closing of alveoli is called atelectotrauma, and positive end-expiratory pressure (PEEP) is important in preventing this type of mechanical injury, which was reviewed in 1967 by Ashbaugh et al. [42]. Biotrauma is the mediator of not only all kinds of mechanical injury following pulmonary injury but also systematic injury [43,44]. One specific low-stretch ventilation strategy was considered to reduce inflammatory factors, such as IL-1, IL-6, IL-8, and TNFα in both plasma and bronchoalveolar lavage [45,46].

The pathobiology features of VILI and ARDS share many inflammatory processes and pathways, including disruption of endothelial barrier integrity and the resulting alveolar flooding, while existing evidence suggests that loss of endothelial cell function plays a central role in the occurrence and development of VILI [47]. One translational research study carried out on mice suggested that the pulmonary endothelial cell is the most responsive type of cell to high tidal volume ventilation, followed by inflammatory activation and the epithelium and macrophages being significantly distinct from the LPS-induced inflammatory process, regarding the activation of the NF-κB pathway [48]. It is also suggested that activated pulmonary monocytes and macrophages and their secreted growth factors contribute to post-VILI recovery [49].

Currently, studies on VILI in animal models have guided researchers to focus mainly on the mechanical injury to endothelial cells, and some researchers have attempted to use DRD to act on endothelial cells to reduce VILI by regulating microtubule stability [50]. However, there are few studies on the impact of VILI on ventilation and blood flow and limited research on the damage to non-pulmonary organs by inflammatory mediators and inflammatory cells [51]. Due to factors such as economic conditions, individual heterogeneity, and the complexity of gene manipulation, research applied to animal models cannot provide enough information.

## 2. Advances in Lung Organoids

Recently, organoid technology has been widely adopted in numerous research scenarios. Advances in regenerative medicine and the advent of induced pluripotent stem cells have enabled the symbiotic progression of somatic cell induction systems and organoid research [52]. Developmental knowledge and induction systems of terminally differentiated cells can be gleaned from multi-cell organoids or spheroids, while various kinds of induced somatic cells can be further used to generate organoids for disease modeling and even clinical applications. On the other hand, lung organoids have made noteworthy contributions toward combating COVID-19. The pandemic has had a grave effect on economic output, yet it has also increased the implementation of organoids in disease modeling [53]. In the upcoming section, we will highlight the progress and generality of lung organoid research and discuss its potential use in ARDS research.

### 2.1. Cell Source and Type

**Figure 1:**
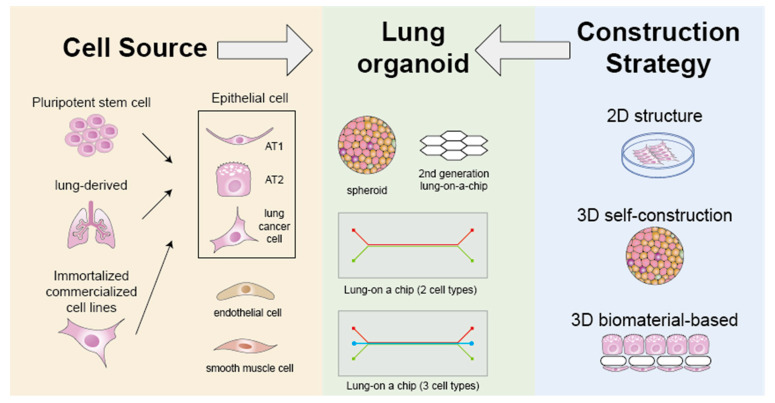
Cellular and assembly strategies of lung organoid construction. This figure illustrates the basic strategy for constructing lung organoids. Based on the research objectives and material availability, appropriate cell sources and construction methods should be carefully selected. Cell sources (left side) include pluripotent stem cell-derived epithelial cells, human or animal tissue-derived primary cells, and commercially available cell lines (could be immortalized or cancer cells), supplemented with other cells used for organoid construction, such as endothelial cells, smooth muscle cells, fibroblasts, etc. Common construction strategies involve direct 2D cultivation, 3D self-assembly, and biomaterial-based 3D assembly. The middle section enumerates several classic lung organoids and their developmental models, such as spheroids and lung-on-a-chip models composed of various cell types. Notably, the innovation in lung-on-a-chip models extends not only to cell diversity but also to the biomaterials separating the cells, as described in the foundational model of second-generation lung-on-a-chip materials by Pauline Zamprogno et al. (upper middle). The depiction of lung organoids capable of mimicking mechanical ventilation is not included in this figure. For insights into this aspect, refer to the detailed description provided by Oliver Guenat et al. in their highly insightful work.

The construction strategy of organoids is strictly dependent on the types and sources of cells adopted. The respiratory tree is entirely composed of the respiratory epithelium, i.e., ciliated cells, goblet cells, basal cells, brush cells, and neuroendocrine cells. There is also circulation system branching along with the epithelium, which is mostly composed of epithelial cells, and smooth muscle cells. Tamas et al. identified four essential cell types crucial for maintaining lung functionality: fibroblast cells, alveolar epithelial cells, endothelial cells, and smooth muscle cells (Figure 1). These cell types encompass both functional cells necessary for normal lung function and structural cells that offer mechanical support [54]. The largest volume of the lung is comprised of an alveolar sac, which is an elaborate sac-like structure composed of epithelial cells surrounded by blood capillaries. Therefore, until now, most lung organoids have been developed based on limited types of epithelial cells and endothelial cells.

When it comes to the cells available for lung organoid construction, three sources can be identified: human pluripotent stem cell (hPSC)-derived cells, patient-derived primary cells, and commercialized cell lines. hPSCs and cell lines are often chosen as alternatives due to the difficulties in obtaining primary cells, making them useful for disease modeling, drug testing, and mechanism studies. While hPSCs provide an almost unlimited source of somatic cells, they are more akin to fetal cells rather than their adult counterparts. Primary cells, attributed to their natural origin, are deemed to possess superior quality, establishing the standard for in vitro differentiation systems. The transplantation of human-derived distal airway stem/progenitor cells stands as a promising therapeutic approach for COPD, signifying their pluripotent nature and therapeutic potential [55]. Nevertheless, their acquisition remains a major impediment to research. Commercialized cell lines are the closest replacement to in vivo and primary cells, and they are the most commonly used type of cell, yet their immortalization process could adversely affect some biological processes.

Green et al. [56] and Huang et al. [57] established a foundational system for the differentiation of the anterior foregut endoderm and lung progenitor cells, providing a valuable platform for subsequent studies. Green and colleagues were the first to demonstrate that the simultaneous inhibition of TGF-beta and BMP signaling, subsequent to endoderm specification, yields a heightened enrichment of the anterior foregut endoderm [56]. Building upon this, Huang and colleagues carefully refined the mouse anterior foregut endoderm differentiation system through a sequential application of molecular inhibitors of BMP, TGF-beta, and Wnt signaling pathways. This optimization enables precise control over the development of lung progenitors. Both in vitro and in vivo experiments have confirmed the multipotency of these lung progenitors. They exhibit the ability to generate various types of lung and airway cells, proving the effectiveness of this optimized differentiation system [57].

AT2-like cells have also been successfully generated through a month-long protocol, recapitulating the development sequence of the distal lung, and the epithelial-only 3D spheres can be maintained in vitro for up to one year [58]. Multiple biological pathways, such as Wnt, BMP, TGFb, FGF, and YAP/TAZ signaling, have been identified as key regulators of lung epithelial cell differentiation in mice [59,60,61,62,63,64,65]. A singular population of AT2 cells may not faithfully recapitulate the local microenvironment of the lung, yet it offers significant advantages, particularly in investigating the intracellular processes of host–pathogen interactions and virus-targeted cellular responses to pathogens. For instance, during the COVID-19 pandemic, Tata’s laboratory rapidly developed a chemically defined culture system that efficiently generated alveolospheres primarily composed of AT2 cells. The expression of the angiotensin-converting enzyme receptor type-2 (ACE2) in these spheres made them an excellent platform for drug screening. Their research promptly elucidated the impact of viral infection on AT2 cell functionality, highlighting its induction of apoptosis. Furthermore, they identified numerous IFN ligands and target genes activated by AT2 cells in response to COVID-19 infection [66]. Of particular interest, Gotoh et al. discovered a subset of SFTPC cells in their alveolar organoids designed for AT2 cell production, which exhibit similarities to primary AT1 cells upon single-cell transcriptome analysis, and the inhibition of canonical Wnt signaling has been shown to promote AT1 cell differentiation [67]. More recently, Burgess et al. employed a combination strategy of LATS inhibitor, Wnt withdrawal, and FGF withdrawal in the differentiation system, resulting in the production of AGER+ cells in over 80% of the NKX2-1^+^ cells [68]. These AT1 induction programs have also been tested in primary AT2 cells. The optimization of various epithelial cell types and the development of an established endothelial induction system [69,70,71] have facilitated the construction of multiple-lineage lung organoids.

Zacharias et al. identified a specific subset of primary AT2 cells, distinguished by the surface marker TM4SF1, as alveolar epithelial progenitor (AEP) cells. AEP cells express most of the AT2 cell markers in both mice and humans but demonstrate significant epigenomic dissimilarity from AT2 cells, with 40% of the genes exhibiting differential chromatin accessibility as determined by ATAC-seq [72]. Clonal alveolar organoid assays demonstrated that AEP cells play a central role in differentiation into AT1 and AT2 cells in response to Wnt modulation, with AEP-depleted organoids exhibiting reduced responsiveness [72]. Furthermore, the proliferation and differentiation of AEPs into AT1 and AT2 cells were observed in the lungs of H1N1 influenza-infected mice, particularly in zones with mild and substantial injury, but not in those that were entirely destroyed. Given the importance of the AEP lineage in lung regeneration and injury repair, numerous studies on lung organoids and stem cells rely heavily on this pioneering investigation. It is well known that primary alveolar cells are challenging to maintain in vitro for extended periods, significantly limiting the investigation of physiological and pathological processes [73]. Luckily, the swift evolution of sequencing technology has led to the establishment of vast amounts of lung cell atlas data in both healthy and diseased states. These data serve as a valuable resource and benchmark for researchers who face challenges accessing primary cells [74,75,76].

### 2.2. Construction Strategy

The structural construction of organoids is an important parameter for determining their modeling potential (Figure 1). Monolayer culture, which involves one or two types of cells, is the most common classical strategy used in this regard and offers advantages like efficiency and cost-effectiveness. As such, it has become an ideal model for genetic modification, high-throughput drug screening, and mechanism studies [77,78]. However, due to the important role of lung-infiltrating immune cells in physiological functions and pathological processes [34,79], as well as the natural polarity of lung epithelial and endothelial cells, monolayer culture systems cannot adequately model more complicated inflammation processes. Furthermore, native cell processes such as spreading, migration, and intercellular crosstalk cannot be well illustrated by monolayer culture [77,80].

In the realm of 3D culture systems, two primary technical approaches have emerged as viable options: self-construction [81,82] and manufacturing on designed materials [83]. These methods hold tremendous promise in terms of elucidating the mechanisms underlying the disease pathology and development processes. The self-construction approach, in particular, mimics the fetal lung development process and includes well-established differentiation systems that enable the transformation of human pluripotent stem cells (hPSCs) into ventral–anterior foregut spheroids. These spheroids can then differentiate into either lung organoids or bud tip progenitor organoids [82,84,85]. Specifically, Alyssa and her colleagues achieved significant advancements in the development of self-assembled lung organoids. Through systematic modulation of key signaling pathways—specifically, Fibroblast Growth Factor (FGF), Wnt, and Retinoic Acid (RA)—they demonstrated the directed differentiation of foregut spheroids. This orchestrated modulation leads to the generation of distinct outcomes: the formation of airway-like structures, with mesenchyme and progenitors embedded or the derivation of bud tip progenitor organoids. The bud tip progenitor organoids exhibit remarkable stability, maintaining their structural integrity for over 120 days, while alternatively showing the potential for differentiation into diverse lung epithelial cell lineages. This methodology showcases a controlled and strategic approach to steer foregut spheroid differentiation, unveiling promising prospects for studying lung development and modeling lung diseases in vitro [82]. However, mechanical ventilation of the resulting hPSC-derived self-constructed lung organoids is not currently feasible due to their low directional and ordered structure, which makes the formation of connected bubble-like structures nearly impossible. As a result, hPSC-derived models are not ideal for a blood–gas exchange study or ARDS for now. Nonetheless, the fetal-like structure of these models makes them an excellent platform for investigating fetal and developmental diseases, although further development and maturation methods are needed.

In the field of material-based lung organoids, Donald and colleagues developed a well-established organ-on-a-chip system for modeling the function of various organs [83]. The first generation of their lung organoid chip consists of a two-chamber system separated by a microporous PDMS membrane, with microvascular endothelial cells and alveolar epithelial cells seeded on opposite sides of the membrane [86]. This model has been utilized to study multiple diseases, including COPD and lung inflammation [87], intravascular thrombosis [88], influenza virus infection [89], and COVID-19 infection [90]. The second generation of their lung organoid-on-a-chip system is capable of mimicking breathing-induced mechanical activity. The organoid model based on this chip system can provide insights into the key components of the pathogenesis of ARDS, and the involvement of immune cells in the blood chamber can theoretically recapitulate most of the key processes of lung injury and repair in ARDS. In contrast, Oliver Guenat and his colleagues developed a different type of chip-based organoid capable of mimicking breathing movement through vacuum stretching [91]. They also optimized the basal membrane in the organoid and tested a stretchable and biodegradable membrane based on collagen and elastin in the lung-on-a-chip system [92]. This new membrane’s flexibility and biocompatibility have outperformed PDMS membranes and opened up possibilities for more advanced and in vivo-like lung organoids.

## 3. Prospects for Future Research

The rapid progression of lung organoids has provided a valuable platform for the research on lung diseases. In addition, the combination of multiple types of cells along with a well-designed culture system have made it possible to simulate complicated diseases in vivo. The pathogenesis and progression of ARDS involve multiple complex biological events, centered on the injury to the endothelium and epithelium, inflammation pathways, and deterioration by inappropriate ventilation strategies. However, methods to simulate and study the progress, combination, and causal effect of these complex biological processes in vivo are currently limited. Inadequate research has impeded the study and treatment of VILI, making it difficult to tell VILI from the diffuse alveolar damage caused directly by ARDS at an early stage.

Lung organoids based on microfluidic chips are excellent models for studying VILI, and currently, many different lung organoid systems are available to simulate mechanical stretch on the endothelium and epithelium during the breathing motion (Table 1). At the same time, the design of the engineered double-chamber model makes it possible to further study gas–blood exchange and characterize inflammatory cells and inflammatory mediators disseminated to the whole body based on blood flow. Currently, research on the occurrence and development of VILI using lung organoids, its influence on multiple organs in the body, gas exchange, and biomarkers related to VILI recognition is still very limited [86,93], which may be partially due to the lack of optimization of air–liquid matching in the organoid. This means that developing an organoid is a time-consuming process. A cut lung injury does not allow a time window. How to demonstrate the interactive relationship between respiratory mechanics and cells in the same time and space context has become an important issue that requires breakthroughs in the future.

At the same time, different types of cells from different sources also provide more possibilities for the application of lung organoids in VILI research. Various types of cells derived from stem cells have superior advantages in terms of cell number, gene manipulation, and abundance of cell type. On the other hand, iPSCs can also be used to study special biological pathways in VILI occurrence in patients with special genetic backgrounds. It is currently unfeasible to obtain lung organoids with air–blood exchange simulation functions solely by using stem cell differentiation, but by combining microfluidic chip-based organoid systems with rapidly progressing stem cell differentiation methods, the related research on lung organoids can develop significantly. So this approach is an interesting one. Of course, this organoid research method can also be applied to other lung disease research models. It is also a good take-off for further research on chronic lung injuries, like COPD.

## Figures and Tables

**Table 1:** Novel design and research findings on lung organoids based on a microfluidic chip.

Year	Design and Features	Main Findings	Ref.
2007	■ Air–liquid interface created by PDMS separated chambers with epithelial cells seeded on both chambers	■ Propagation and rupture of liquid plugs lead to Epi injury by mechanical stresses	[94]
2010	■ Air–liquid interface created by PDMS separated chambers with epithelial cells and endothelial cells seeded on each chamber ■ Enclosed side chamber for the creation of mechanical stretching force	■ Cyclic breathing in a lung-on-a-chip system increased inflammation and oxidative stress, indicating that breathing can worsen toxic responses to environmental particles	[86]
2011	■ Specialized design to study ventilator-induced lung injury (VILI), including bio-inspired respiration motion, flexible PDMS membrane, automated actuation of respiration profile, with only epithelial cell (A549) seeded in the single chamber	■ Mechanical stresses lead to epithelial cell death in the pathogenesis of VILI	[93]
2014	■ Air–liquid interface and three chambers separated by nanoporous membranes with primary Epi, Fb, and EC seeded on each chamber	■ The novel multicompartment device with a well-designed membrane bonding process and co-culture media is available to support organoids with primary human cells	[95]
2015	■ A robust and easy-handling system with bio-inspired respiration motion ■ Air–liquid interface created by PDMS separated chambers with primary epithelial cells and HUVEC seeded on each chamber	■ Increased secretion of IL-8 and increased metabolic activity from epithelial cells was detected after mechanical stretch. No significant changes were observed in death rates in primary epithelial cells	[91]
2021	■ Air–liquid interface separated by a biological, stretchable, and tunable membrane made of collagen and elastin with Epi and EC seeded on each side	■ Novel membrane developed for the next generation of lung organoids with potential	[92]

## Data Availability

Not applicable.
